# Characterization of an iron oxide nanoparticle labelling and MRI-based protocol for inducing human mesenchymal stem cells into neural-like cells

**DOI:** 10.1038/s41598-017-03863-x

**Published:** 2017-06-15

**Authors:** Chen-Wen Lu, Jong-Kai Hsiao, Hon-Man Liu, Chung-Hsin Wu

**Affiliations:** 1 Department of Life Science, National Taiwan Normal University, Taipei, 10677 Taiwan; 2 Department of Medical Imaging, Taipei TzuChi Hospital, The Buddhist TzuChi Medical Foundation, New Taipei City, 23142 Taiwan; 3 Department of Medical Imaging, National Taiwan University Hospital, Taipei, 10048 Taiwan

## Abstract

The aim of the current study was to develop an iron oxide nanoparticle (ION) labelling and magnetic resonance imaging (MRI)-based protocol to allow visualization of the differentiation process of mesenchymal stem cells (MSCs) into neural-like cells (NCs) *in vitro*. Ferucarbotran, a clinically available ION, which can be visualized under MRI, is used for tracking cells implanted *in vivo*. The NCs were verified morphologically and histologically by light microscopy, and their functions were verified by measuring their action potentials. Conformational conversion of axon-like structures was observed under light microscopy. These NCs exhibited frequent, active action potentials compared with cells that did not undergo neural differentiation. The labelling of ION had no influence on the morphological and functional differentiation capacity of the MSCs. We conclude that the MSCs that were differentiated into NCs exhibited *in vitro* activity potential firing and may be used to replace damaged neurons.

## Introduction

Mature neurons do not replicate, which limits their capacity for tissue repair in many conditions such as ischaemic stroke, spinal cord injury, traumatic brain injury, or neurodegenerative diseases^1–3^. Replacing damaged neurons via tissue engineering is theoretically possible in such conditions. Advanced cell imaging is required for administering stem cell therapy via tissue engineering techniques. Stem cell therapies can be potentially used to treat neurological diseases, either for replacing lost neurons, restoring neural circuits or as paracrine-mediated therapies^4–6^. Human mesenchymal stem cells (hMSCs) are multipotent cells that differentiate into bone, osteocytes, cartilage cells, adipocytes and neurons^5, 7^. The differentiation potential of hMSCs into ectodermal cells^8^, such as astrocytes, neurons and oligodendrocytes, has therapeutic potential for neurological diseases^9–11^. Tracking transplanted cells *in vivo* provides direct real-time information on the cell migration, homing, division and/or differentiation, and survival of transplanted cells. Magnetic resonance imaging (MRI) is an excellent tool for studying the fate of transplanted stem cells *in vivo* because it is non-invasive and inherently offers high spatial resolution, the absence of radiation and unlimited tissue penetration depth. In addition, successful monitoring and tracking of stem cells labelled with iron oxide nanoparticles (IONs) has been reported^4^.

Iron oxide nanoparticles have been widely used as clinical contrast agents in MRI for the detection of liver tumours^12, 13^. ION can be internalized into neuron progenitor cells and visualized by MRI for up to 7 days^14^. Once ingested by macrophages or the reticuloendothelial system such as Kupffer cells, ION are metabolized, and the iron core is recycled into the tissue iron pool for the synthesis of haemoglobin. The remainder of the nanoparticle shell, which is primarily composed of sugar-related polymers, is excreted by the kidneys. Our previous study on hMSCs, which were successfully labelled with ION, revealed no significant change to cellular behaviours, such as viability, mitochondrial membrane potential changes or differentiation capacity^15^.

The primary aim of the current study was to develop an MRI-based assay for assessing and comparing the labelling efficiency of ION in hMSCs and hMSC-derived neural-like cells (NCs). The secondary aim was to evaluate and compare the intracellular distribution, cellular toxicity and cell behaviour of the hMSCs and hMSC-derived NCs after ION labelling.

## Results

### Differentiated human MSCs exhibited neural-like morphology and neuron markers: Directly labelling hMSCs and NCs with ION

**Figure 1:**
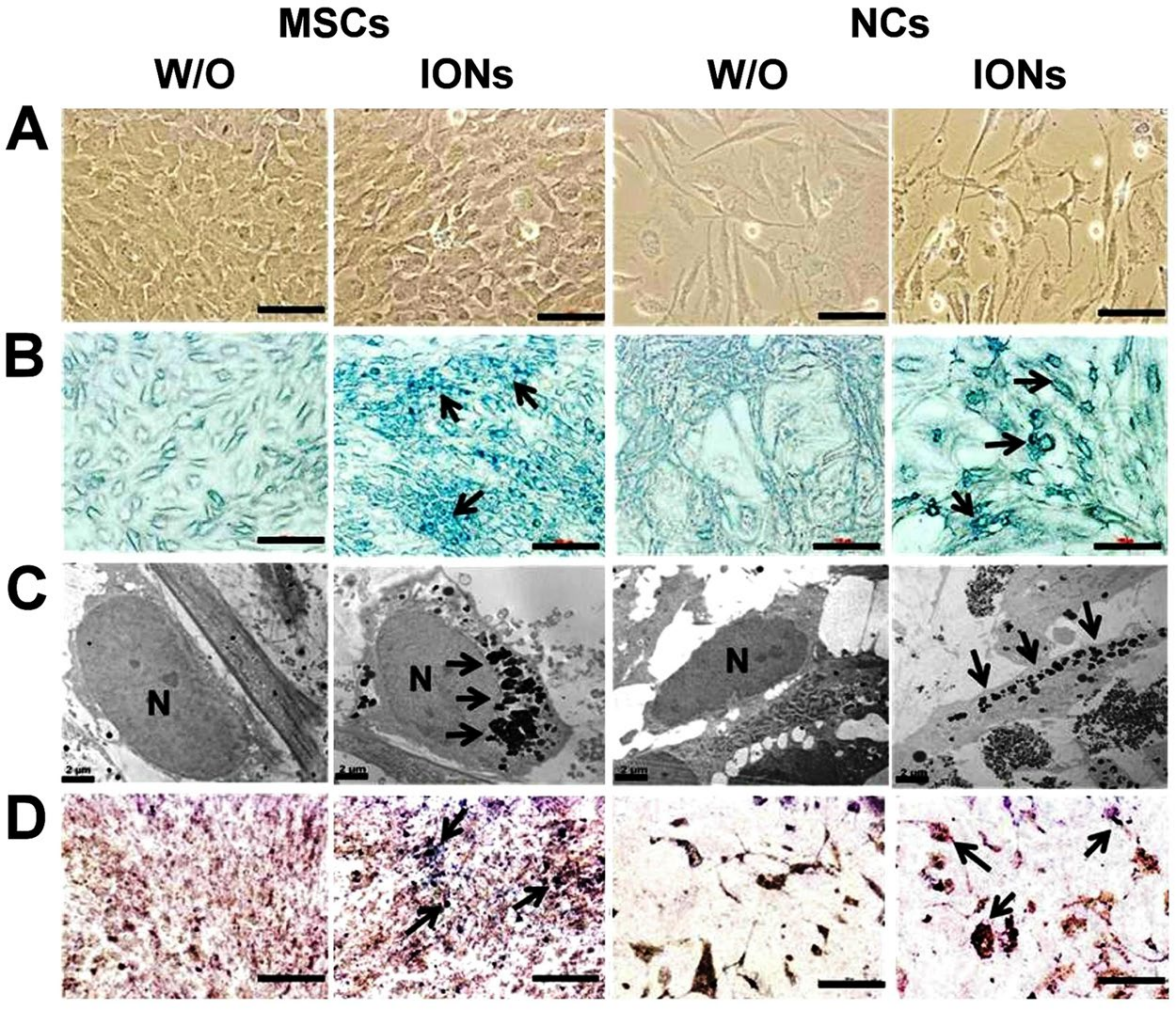
Comparison of hMSCs differentiation capacity into NCs with or without (w/o) ION. Light microscopic image (**A**), Prussian blue staining (**B**), TEM image (**C**) and co-staining with PTAH and Prussian blue (**D**). The blue and black dots indicated by black arrows are ingested ION.

**Figure 2:**
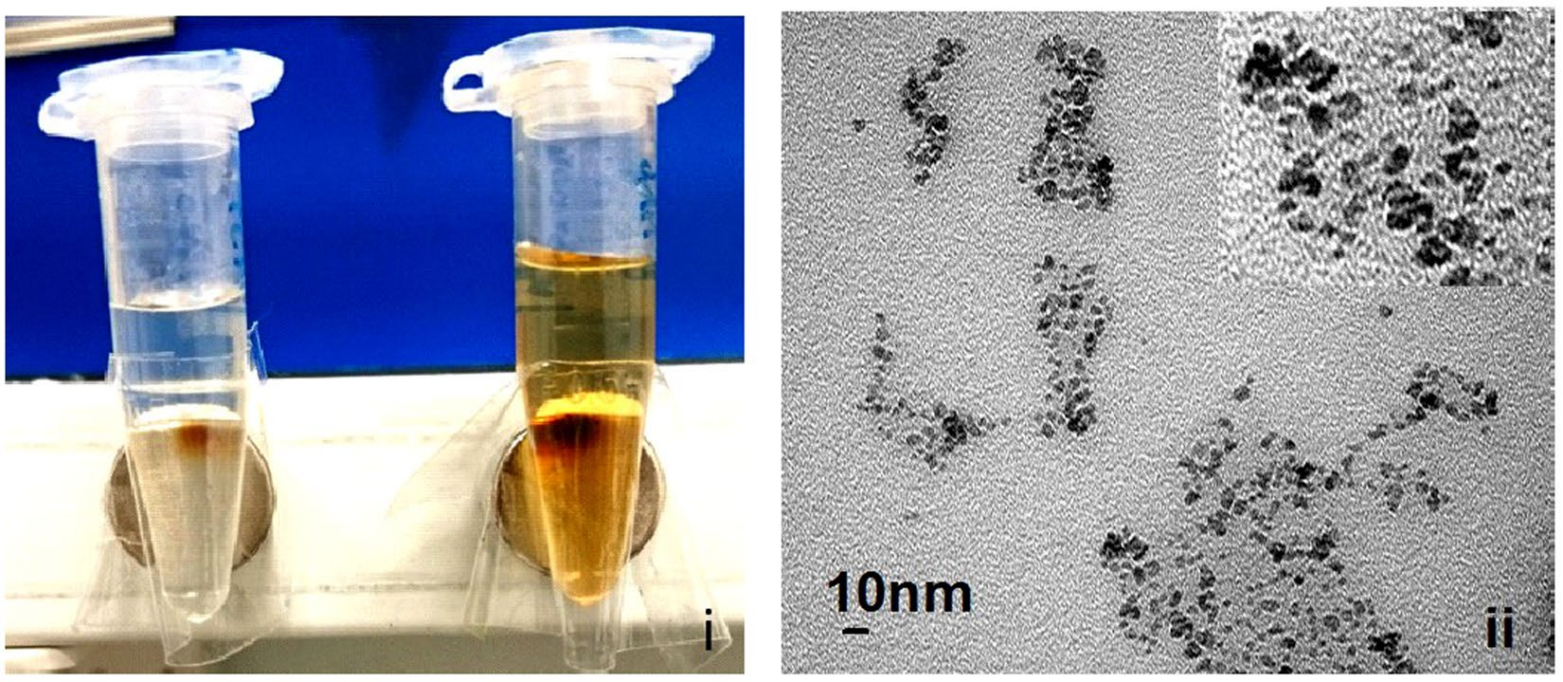
TEM images of ION (Resovist, ferucarbotran). Different concentrations of Resovist solution (i) colour images; left: 10 μg/ml, right: 100 μg/ml) magnetized using a permanent magnet. Particle size was 45–60 nm (ii).

To investigate the *in vitro* differentiation of hMSCs into NCs, hMSCs were incubated in neurogenic induction medium (NIM) for NCs differentiation. Compared with undifferentiated MSCs, NCs exhibited dendrite-like features of long spikes extending into other adjacent cells (Fig. 1A, arrowhead) and lower cell densities (Fig. 1A and B). There was no morphological difference between ION-labelled and unlabelled cells under light microscopy. Both hMSCs and NCs incubated with ION had blue dots precipitated inside the cytoplasm, whereas unlabelled hMSCs and NCs did not have blue dots (Fig. 1B). TEM images also revealed the presence of internalized ION within the organelles of hMSCs and NCs incubated with ION (Fig. 1C). NCs differentiation was further verified by phosphotungstic acid haematoxylin (PTAH) staining. Additionally, co-staining with Prussian blue revealed iron precipitates inside the cytoplasm. Thin and long dendrite-like structures stained in brown were observed in the NCs. By contrast, cells without neural induction exhibited no axon-like structures, and the cytoplasm was not stained. ION-labelled MSCs and NCs exhibited blue precipitate inside cells (Fig. 1D). TEM imaging of the ION structure revealed an inner layer iron-oxide core (Fe_3_O_4_, dark black colour) and a non-magnetic outside layer coated with carboxydextran (grey colour) (Fig. 2).

**Figure 3:**
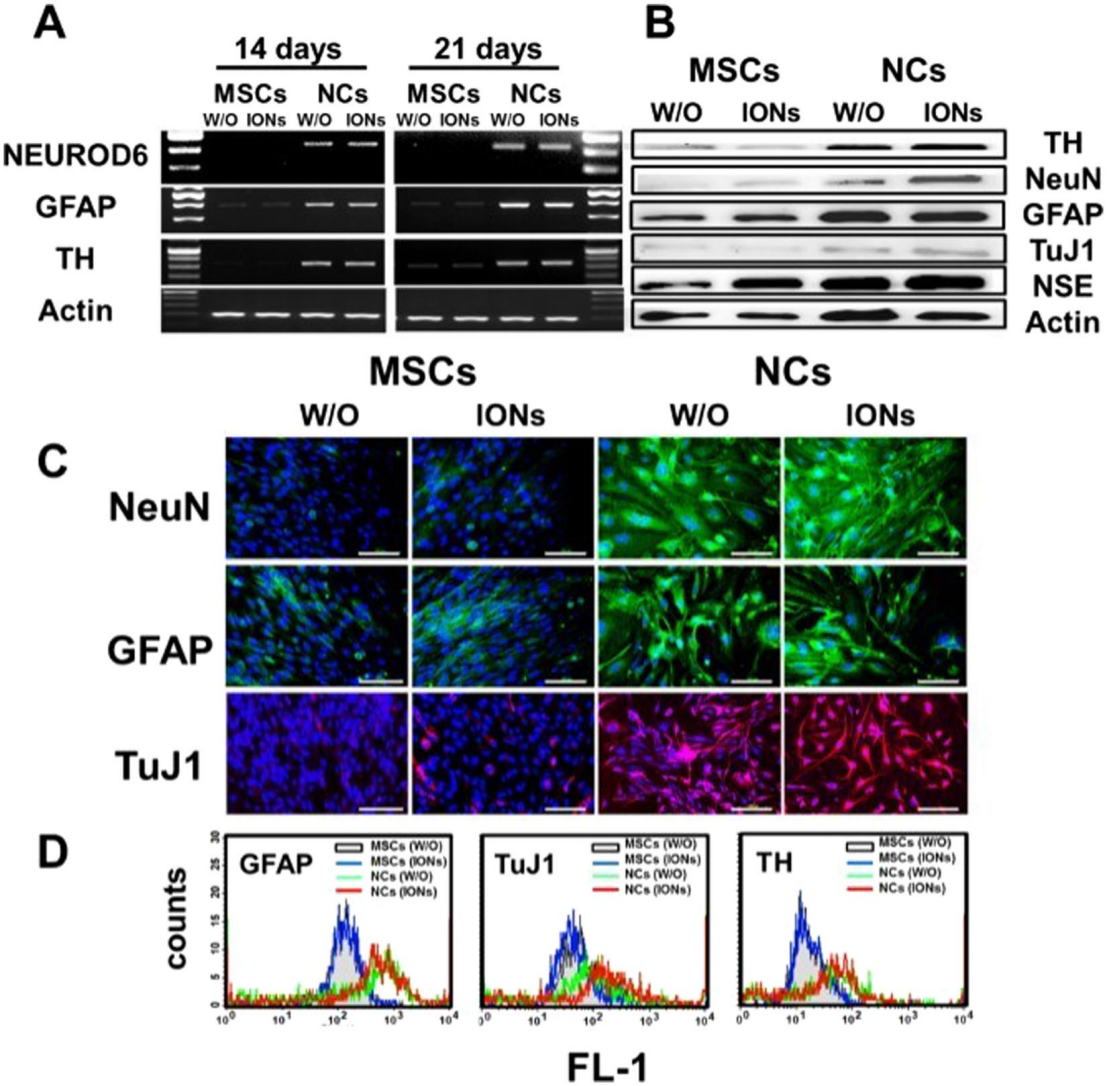
Characterization of neural differentiation markers in hMSCs treated with or without neural induction medium after ION labelling. (**A**) Comparison of neural marker expression (GFAP, TH, and NEUROD6) by RT-PCR after induction of neural-like cell differentiation with or without ION labelling. (**B**) Western blot analysis showing that hMSCs incubated with NIM could be differentiation into NCs and increase the protein expression of TH, NeuN, GFAP, TuJ1 and NSE. Actin as an internal control. (**C**) Immunofluorescent staining of h-NeuN and GFAP in green, TuJ1 in red, and nuclei in blue (DAPI). (**D**) Flow cytometry analysis of GFAP, TH and TuJ1 of the hMSC-derived neural cells with or without ION labelling.

The differentiation of NCs from hMSCs were further evidenced by several neural molecular markers at both the mRNA and protein level (Fig. 3). RT-PCR results demonstrated the expression of glial fibrillary acidic protein (GFAP), tyrosine hydroxylase (TH) and NEUROD6 genes at 14 and 21 days after NIM incubation. The mRNA expression of GFAP, TH and NEUROD6 were significantly elevated in the NC differentiation group regardless of ION labelling. However, no differences in GFAP, TH and NEUROD6 mRNA expression were observed in the hMSC groups (Fig. 3A).

Neuron-specific protein markers GFAP, NeuN, TuJ1 and TH were weakly expressed in undifferentiated MSCs. However, the protein levels of these markers were dramatically expressed in NCs (Fig. 3B). With immunofluorescent staining, the expression of GFAP, NeuN and TuJ1 were visualized as strong fluorescent signals inside the cytoplasm after NIM treatment in NCs groups. ION labelling did not alter the expression of the GFAP (stained in green), NeuN (stained in green) and beta-III tubulin (TuJ1, stained in red) (Fig. 3C). Expression levels of GFAP, TuJ1, and TH were also confirmed after neural-like cell differentiation by FACS analysis (Fig. 3D). The NCs had a significantly higher mean expression of NCs / MSCs (GFAP: 958/168, TuJ1: 707/87, and TH: 637/40) compared with hMSCs with or without (w/o) ION treatment.

### Electrophysiological function

**Figure 4:**
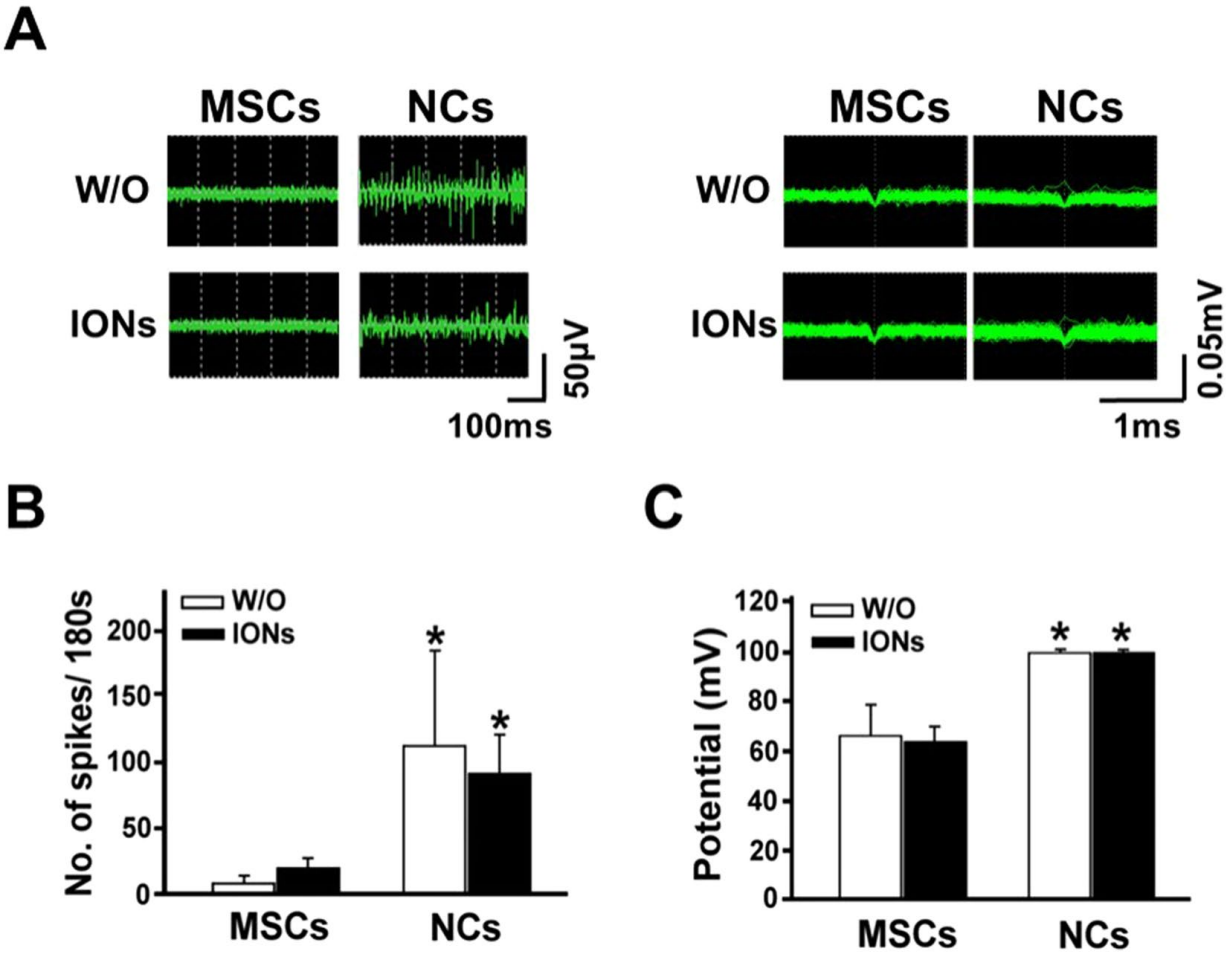
Action potentials of hMSCs, NCs with or without ION labelling. (**A**) Quantitative analysis of the action potentials (mV, millivolt; ms, milliseconds). (**B**) The number of spikes are reported. (**C**) Both action potential amplitude and the number of spikes increased after neural-like cell induction. ION labelling had no influence on the action potential amplitude or number of spikes.

The electrophysiological characteristics of ION-labelled MSCs and NCs are shown in Fig. 4. The ability acquired by NCs differentiated from MSCs to generate spontaneous firing activity patterns was not altered after labelling with ION (Fig. 4A). The quantitative results and spike frequency of the cells with neural-like morphologies indicated the active membrane properties of the cells, as shown in Fig. 4B.

There was no significant difference between NCs with or without ION labelling. Moreover, the voltage of each action potential signal was higher in the cells in the NCs group (90 mV) than in cells that did not undergo NCs differentiation (50–60 mV) (Fig. 4A,B) Labelling with ION did not alter the voltage amplitude of the spontaneous firing activity. By contrast, MSCs did not express significant spontaneous firing activity patterns. In the 180-s observation period, the cells that differentiated into neural-like cells generated more spikes (80 times/180 s) than the cells that did not undergo differentiation (10–30 times/180 s). Taken together, these data demonstrated that NCs differentiated from MSCs have key features of functional neurons with the long-term ability to generate spontaneous firing activity patterns.

### *In vitro* determination of ION uptake by MRI, inductively coupled plasma mass spectrometry (ICP-MS) and flow cytometry

**Figure 5:**
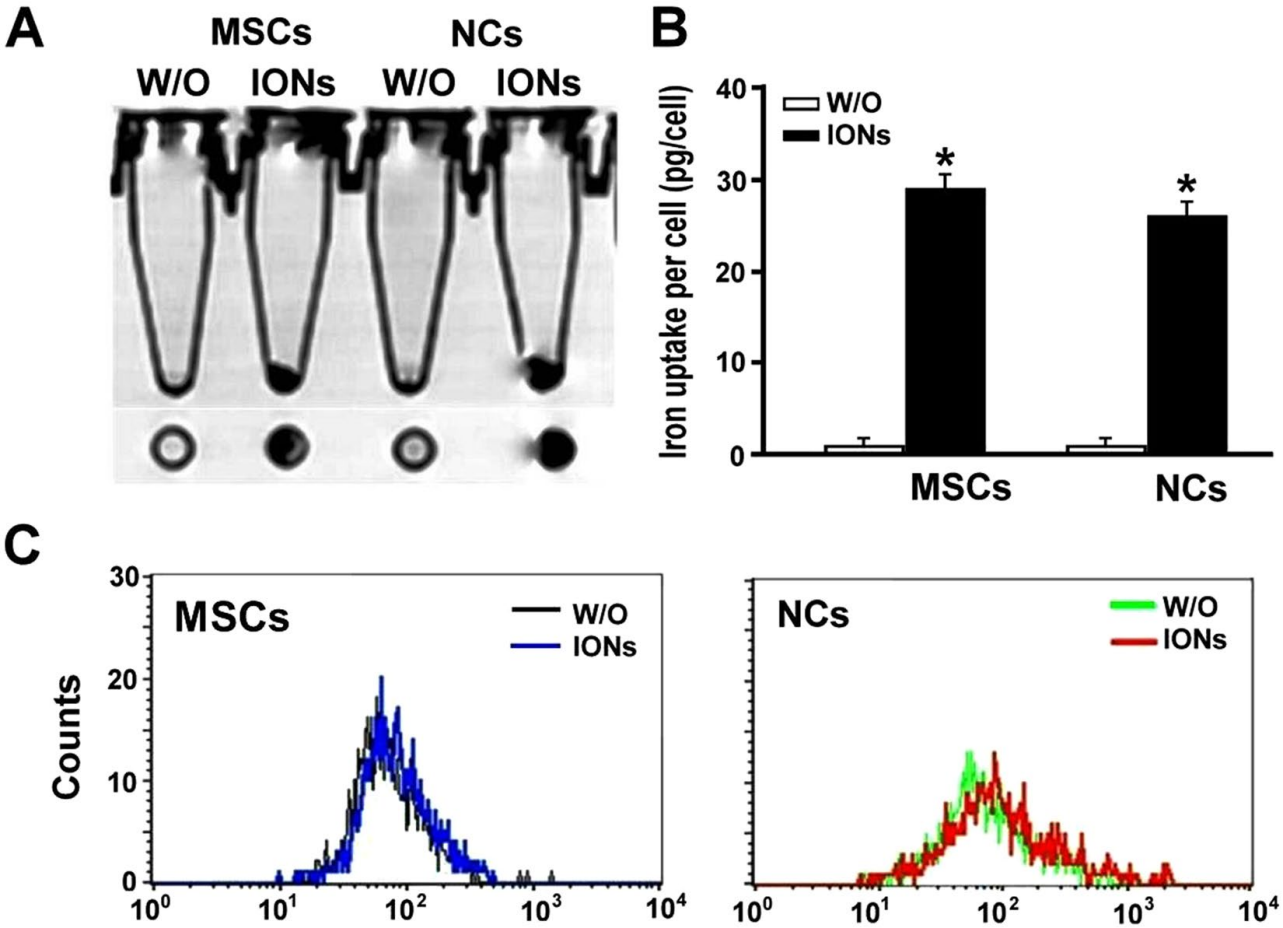
Quantification of iron content after labelling with or without ION before and after induction of neural-like cell differentiation. (**A**) *In vitro* MRI, (**B**) ICP-MS, and (**C**) SSCs based on flow cytometry of each group. We observed increased iron content in ferucarbotran-labelled cells and slightly increased SSCs in flow cytometry, indicating increased granularity in ION-labelled cells. *In vitro* MRI also demonstrated that the labelling efficiency could be visualized by clinical MRI.

We next examined whether the ION-labelled cells can be detected under non-invasive MRI. Under T2-weighted images, ION-labelled NCs and MSCs showed dark dots at the bottom of the test tube, whereas no dark signal was detected in cells without ION labelling (Fig. 5A). The iron content of the cells was determined using ICP-MS (Fig. 5B). The intracellular level of iron was significantly greater in MSCs and NCs treated with ION (29.2 ± 1.5 pg/cell and 25.9 ± 2.0 pg/cell, respectively) than in untreated MSCs and NCs (0.38 pg/cell and 0.93 pg/cell, respectively). Cell granularity determined by flow cytometry showed more side scatter counts (SSCs) in the ION-treated cells than in the untreated cells (Fig. 5C).

### Cell behaviour

**Figure 6:**
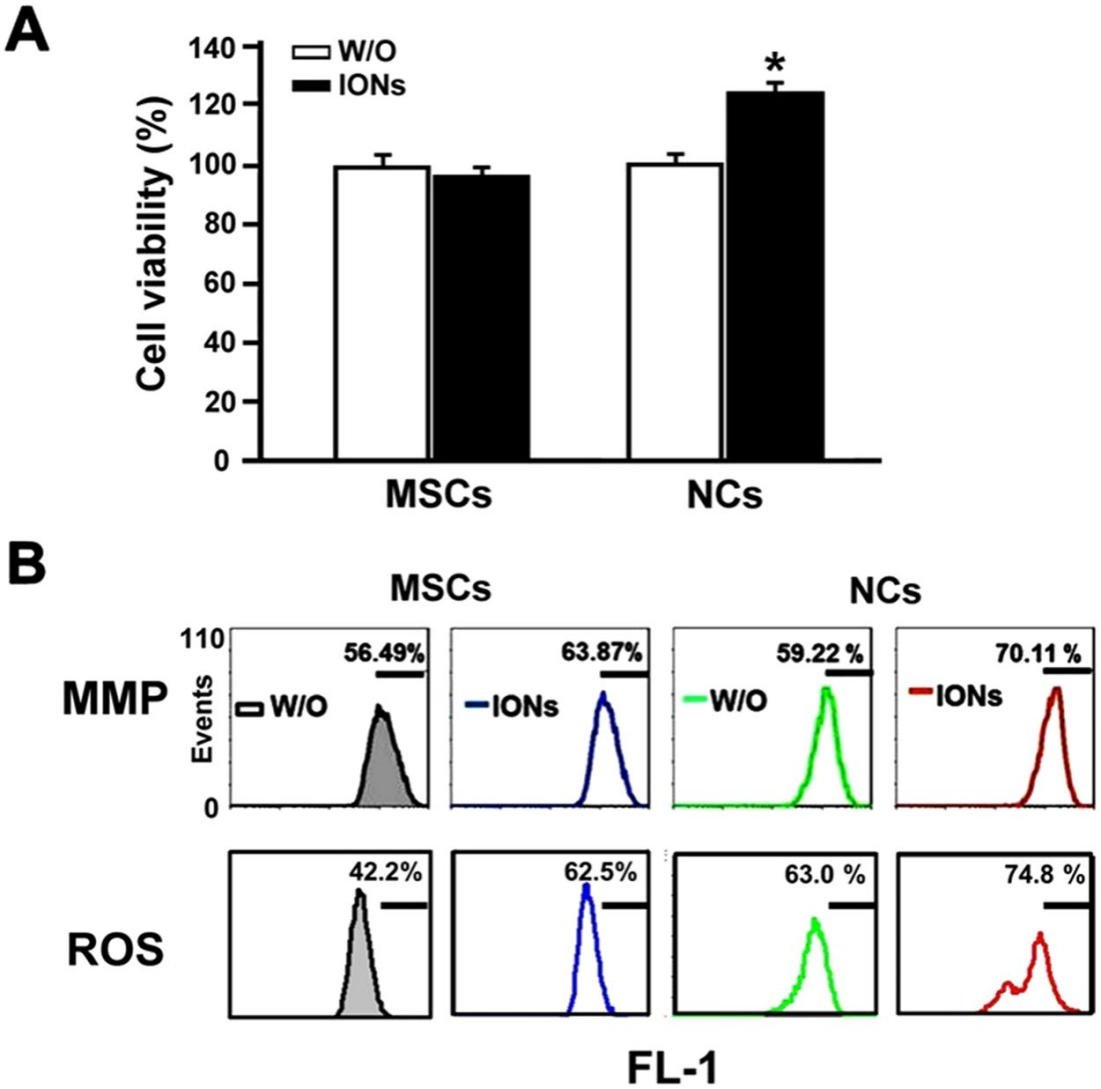
Measuring cell behaviour using three different assays. (**A**) Cell viability (MTT) assay, (**B**) mitochondrial membrane potential (MMP) and reactive oxygen species (ROS) assay (FL-1, fluorescent mean intensity). Human mesenchymal stem cells with or without 21 days of differentiation induction were treated with or without 100 μg/mL ION for 24 hours.

Cytotoxicity testing of ION-treated cells was verified by using 3-(4,5-dimethylthiazol-2-yl)- 2,5-diphenyltetrazolium bromide (MTT), mitochondrial membrane potential (MMP) and reactive oxygen species (ROS) production assays (Fig. 6). The MTT assay showed a significant increase in formazan formation in ION-labelled NCs but not in ION-labelled MSCs (Fig. 6A). There was no significant alteration in the MMP of hMSCs and NCs labelled with IONs. ION-labelled hMSCs and NCs displayed elevated ROS production compared to unlabelled hMSCs and NCs (Fig. 6B).

## Discussion

In this study, we demonstrated that ION labelling does not affect the differentiation capability of hMSCs to NCs. Cellular functions, including morphology, viability, oxidative stress, and mitochondrial member energization, of ION-labelled hMSCs and NCs were intact. IONs induced no significant difference in mRNA, protein content, neuron-specific protein marker expression, spontaneous firing activity patterns, or intracellular iron levels in NCs.

The clinically used ION, ferucarbotran, has a high uptake capability by stem cells and can be detected using the 1.5 T MRI at the single-cell level^15–17^. Our study revealed a slight increase in cell growth after ION labelling possibly because the cell cycle is involved after intracellular Fe release from lysosomes^18^. The ROS of labelled cells were slightly increased due to the increase in H_2_O_2_ in the cytoplasm^19^. These results are consistent with the findings of Chen *et al*.^14^.

Similar to the previous findings^20, 21^, we found no influence of ION uptake on the MMP.

Different sizes of supraparamagnetic ION have been used in labelling and tracking neural-like cells during differentiation using MR imaging methods. Such ION include ultrasmall superparamagnetic iron oxide (USPIO) particles (≤30 nm in diameter), superparamagnetic iron oxide (SPIO) particles (30–200 nm in diameter), and micron-sized superparamagnetic iron oxide (MPIO) particles^22^. After intravascular injection, USPIO enters the capillaries, then the matrix, and ultimately arrives at the lymph nodes, but most SPIO accumulates in the reticular endothelial system^23^. Crabbe *et al*. compared the labelling efficiency of three different stem cell populations [mouse embryonic stem cells (mESCs), rat multipotent adult progenitor cells (rMAPCs), and mouse mesenchymal stem cells (mMSCs)] with three different (ultra)small superparamagnetic iron oxide [(U)SPIO] particles (Resovist, Endorem, Sinerem). The labelling efficiency with Resovist and Endorem significantly differed among stem cells. They found that the minimum cell density that needed to be detected by the MR imager was 75 cells/μl in Sinerem® (USPIO, 20 nm), and 5 cells/μl in Endorem® (SPIO, 80–150 nm) and Resovist® (SPIO, 60 nm). After labelling with Resovist, rMAPCs can still express the neuroprogenitor genes Sox2 and Pax6. They also observed migration to the injured area 3 weeks after implanting 10,000 Resovist-labelled rMAPCs in the bilateral striatum^24^. Based on this previous research, we sought to label MSCs and NCs with SPIO, i.e., Resovist. Many studies have shown no morphological changes in MSCs, neural stem cells (NSCs), or neural progenitor cells (NPCs) labelled with MPIO, and all cell types expressed TH, TuJ1, and nestin after MPIO labelling. As MPIO particles are larger, they are more easily detected, especially at the single-cell level. Another advantage of MPIO particles for cellular tracking is that they have a longer half-life than (U)SPIO particles in the body. Evaluation of intracellular labeling with micron-sized particles of iron oxide (MPIOs) as a general tool for *in vitro* and *in vivo* tracking of human stem and progenitor cells^25^. However, (U)SPIO particles can be metabolized over the long-term, whereas MPIO particles with a size >100 nm cannot.

Ferucarbotran is an ION with a particle size of approximately 60 nm and is composed of an iron oxide core coated with carboxydextran to prevent nanoparticle aggregation. The iron oxide is metabolized and reused as a haemoglobin component. Carboxydextran is degraded by lysosomes^15^. A recent study demonstrated that, after exposure of human neuroblastoma cells to 10 μg/ml of 10 nm ION for 24 h, obvious neurotoxicity, including decreased cellular dopamine, increased ROS, increased neural α-synuclein and activated tyrosine kinase c-Ab1 expression, and inhibition of cell-proliferation, were observed in neural cells^26^.

Another study investigating the size and relationship of ION retention in the central nervous system concluded that 40-nm ION possess more evident detention properties in the CNS than 280-nm ION^27^. Another type of ION, the Molday ION, have also been introduced, which are new ultra-small superparamagnetic ION with a magnetic core and hydrodynamic sizes of approximately 8 and 35 nm, respectively, conjugated with Rhodamine-B (Rh-B) (2 fluorophores per particle). The Molday Rhodamine-B ION significantly reduced the survival, proliferation, and differentiation rate of neural stem cells, and upregulated the immune response in recipient animals in a concentration of 50 μg/mL^28^. Such characteristics are not suitable for use as a labelling agent. We speculate that this might be the result of the very small sizes of the particles, the coating material and its fluorophore ligands. These support the possibility that ferucarbotran may be one of the best ION for labelling cells for studies of the nervous system.

We selected Resovist ION because Resovist is a clinically used MR contrast medium approved by the FDA. Internalization can be performed without any transfection agent. Resovist has several advantages such as its good biocompatibility, low toxicity, and excellent stability^29^. Using Resovist labelling, studies of rabbit MSCs revealed that it induced no changes in morphology, it had no effect on neural differentiation, and the protein expression levels of the mature neuron markers TuJ1 and NSE were maintained^30, 31^. These findings are similar to our results reported here. Another report using human amniotic membrane-derived MSCs (hAM-dMSCs) showed no expression of GFAP protein, although there was no resulting morphological change. This finding might be due to the fact that glial formation cannot be detected in a relatively short period of incubation during neural-induction differentiation (7 days) or that a different source of MSCs was used^29^.

To improve biocompatibility and biodistribution and to prevent precipitation and agglomeration under physiological conditions, SPION particles are coated with an amphiphilic layer of polymers. These polymers can be hydrophilic and protect the iron oxide core from degradation^32^. Resovist is coated with carboxydextran, which is a type of natural polysaccharide with a net charge, water solubility, biocompatibility, and biodegradability and is enriched with hydroxyl groups, which have been reported to interact with iron oxide via hydrogen bonding.

In addition, poly(L-lysine) (PLL), a positively charged peptide, can be used to facilitate cellular internalization. Ferumoxides-PLL complex-labelled MSCs does not alter biochemical or haematological measures of organ function^33^. Albukhaty *et al*. reported that a mixture of 25 μg/0.75 μg ml SPIO/PLL enhanced cellular activity after differentiation^34^. Ke *et al*. labelled bone marrow stroma cell-derived neural stem cells (BMSC-D-NSCs) with PLL-coated Feridex and found no morphological changes after differentiation; NSC and GFAP expression were also preserved. After autologous implantation into the monkey brain, BMSC-D-NSCs were still locally detected by MRI as black dots, and they could express NSE protein according to immunohistochemistry assays^35^. Delcroix and associates used another negatively charged material, 1-hydroxyethylidene-1.1-bisphosphonic acid (HEDP)-coated SPIO particles, to label rat MSCs and found no morphological changes after differentiation and preserved TuJ1 and NeuN expression^36^.

Chen *et al*. labelled mouse NSCs with hydrogen-terminated ultra-nanocrystalline diamond (H-UNCD) and found that it causes spontaneous neural differentiation of NSCs. They claimed that the characteristics of wear/corrosive resistance in this crystalline structure might improve cellular adhesion and extension. This was proven in their scanning electronic micrograph study, which showed that the filopodia of NSCs extended more than 100 nm and that the expression of neuron markers such as GFAP and TuJ1 was increased according to immunofluorescence staining^37^. As the underlying mechanism, they proposed that the crystalline structure can increase the linkage between NSCs and cytokine or integrin release, which is not related to the hydrophobicity.

The above studies provide insight into some particular surface structures, such as UNCD, that might have an impact on the differentiation of NSCs, but the charge does not seem to be an important factor.

After labelling hMSCs with ferumoxides, another type of ION, the iron content inside the hMSCs decreased upon cell division^38^. Both decreased iron content and loss of cellular granularity are correlated with a decrease in the long-term detectability of cells by MRI^39^. In a clinical MRI study^40^ of traumatic brain injury patients in which autologous neural stem cells were labelled with ferumoxides and transplanted into the brain injury area, strong signal changes were detected from days 1 to days 14. After 2 weeks, the cells began to move around the damaged area, and the signal weakened. No signal was detected until the 7^th^ week, which may be related to the fact that the calibrated neural stem cells had moved and were scattered in relation to the localization region. Consequently, the detectability of ION-labelled neurons is dependent on the initial iron oxide labelling concentration and the speed of cell division. Higher initial iron concentration might influence cell behaviour that is averse to neuron repair. Our study shows that up to 100 μg Fe/mL for incubation is still within the safe range and has little influence on cell viability and differentiation. The intracellular levels of iron in hMSCs and NCs treated with ION were 29.2 ± 1.5 pg/cell and 25.9 ± 2.0 pg/cell, respectively, in the current study, which are comparable to our previous report (23.4 pg/cell in ION of 100 μg Fe/mL)^15^ and slightly higher than the levels reported in another study (15–20 pg Fe/cell in ION of 20 and 30 μg Fe/ml)^24^. This suggests that MR imaging using our ION-labelling protocol can successfully localize the stem cells and can be used to track their persistence and migration over time in animal models. Although a previous report claimed nearly 100% labelling efficiency of ION by Prussian Blue iron stain alone, no quantitative evidence was provided to support the conclusion^31^.

The implantation of MSCs into rats that underwent spinal cord injury or stroke improved their performance in previous studies. However, MSCs implanted into the rat brain do not differentiate into functional neurons^41–44^. Similarly, the implantation of adult NCs failed to improve cell function and migration, which are critical in neuron repair^45^. However, we showed that the induction of MSCs by growth factors is morphologically effective. Moreover, the migration of cells can also be monitored by non-invasive MRI methods. The combination of growth factor induction and ION labelling will facilitate future research in neuron tissue engineering.

NSCs derived from rabbit BM-MSCs did not affect the morphology of neurons and expressed specific neuroprotective proteins (NSE, MAP) after labelling with ferucarbotran^31^. Whole-cell patch-clamp recordings showed that these NCs exhibited electrophysiological activity. Our labelled hMSCs differentiated into NCs that exhibited significant levels of mature neural biomarkers, including observable dendrites and spontaneous firing activity patterns.

Neural function still cannot be restored in patients suffering from dementia, Parkinson’s disease, and stroke even despite aggressive treatment methods. The treatment of these disorders with conventional methods or medication can partially relieve symptoms. Furthermore, the spontaneous generation of neural cells after brain injury is limited. Cell therapy can provide a chance to regain neuronal function by replacing dead or degenerative neurons with newly differentiated cells^46–48^.

The induction of pluripotent stem cells that originate from human fibroblast has drawn much attention in cell replacement therapy due to the autologous origin of the fibroblasts. However, the tumorigenicity found in phase I clinical trials has limited the applications of this technique^49^. However, hMSCs have extensive differentiation capacity without inducing tumorigenicity, they are easy to propagate, and they exhibit immunomodulatory properties that are beneficial in damaged brain tissue^50^. However, whether these differentiated NCs retain their immunomodulatory capacity should be further evaluated.

Induced neural- like cells implanted into the spinal cords of rats that underwent spinal cord injury improved neurogenesis in rat models and improved function in models of Parkinsonism^51, 52^. Such induced neural cells exhibited higher levels of neural markers^53, 54^. It is important to be able to confirm whether hMSCs can express the proteins of nerve cells before or during implantation *in vivo* and in pre-clinical trials. In our study, the ION-labelled NCs expressed multiple mature neural protein markers and produced many NCs *in vitro*, and thus, they could possibly be used to replace irreparably damaged nerve cells.

## Conclusion

We established an ION-labelling and MRI-based protocol for studying neural stem-like cells differentiation and hMSC-derived NCs. All the derived NCs exhibited significant mature neural biomarkers, including observable dendrites and spontaneous firing activity. In ION labelling, the process of implantation and cell migration can be traced by MRI, which is ideal for the analysis of implanted cells in damaged regions and can shed light on cell therapies applied to the central nervous system.

## Materials and Methods

All experimental procedures for hMSC culture were approved by the Committee on Biological Research of National Taiwan Normal University and implemented under the guidelines of the Committee.

### Cell culture

#### Human mesenchymal stem cell culture

hMSCs that had been immortalized through the transfection of human telomerase reverse transcriptase (hTERT) with human papillomavirus E6 and E7 were a gift from Li-Horn (1). The culture medium (CM) consisted of high-glucose Dulbecco’s modified Eagle’s medium (DMEM) (Gibco, BRL, Grand Island, NY, USA), supplemented with 10% foetal bovine serum (FBS; HyClone, Logan, UT, USA), 4 mM L-glutamine, 100 U/mL penicillin, and 100 μg/mL streptomycin (Gibco, NY, USA) (Gibco BRL, Grand Island, NY).

### Neurogenic differentiation of human mesenchymal stem cells

HMSCs labelled with or without ION were plated at a density of 3 × 10^4^ cells/cm^2^ in 6-well culture plates. When the cells were confluent, neurogenic differentiation was initiated by NIM culture containing DMEM (Gibco BRL, Grand Island, NY) with 10% FBS, 10 ng/mL basic-fibroblast growth factor (b-FGF), 10 ng/mL epidermal growth factor (EGF), 10 ng/mL brain-derived growth factor (BDGF, R&D Systems, Minneapolis, MN, USA), 1 ng/mL platelet-derived growth factor (PDGF, R&D Systems, Minneapolis, MN, USA), 0.5 mM all-trans retinoic acid (ATRA), 1X N1 medium supplement solution (Sigma-Aldrich Co., St. Louis, MO, USA), 2 mM valproic acid ((Sigma-Aldrich Co.), and 10 μM forskolin and 1 M hydrocortisone (Sigma-Aldrich Co.). The induction medium was changed every 2~3 days for 21 days.

#### ION labelling

For incubation with or without ION, ferucarbotran ION (Resovist®, Bayer Pharma AG, Berlin, Germany) were added to the culture medium at 100 μg Fe/mL. After 24 hours incubation at 37 °C in 5% CO_2_, the cells were further evaluated using Prussian Blue staining (Sigma-Aldrich Co.) and MRI. Examination of neural protein expression through flow cytometry, immunofluorescence, Western blotting and RT-PCR analysis were also conducted.

### Morphological analysis

#### Transmission electron microscopy

We used permanent magnets to observe the concentration of iron oxide ions by placing 10 µg/ml and 100 µg/ml ION in a centrifuge tube. The intracellular 100 µg/ml ION uptake by cells was confirmed by transmission electron microscopy (TEM). 1 × 10^4^ labelled or unlabelled cells were cultured in a plastic chamber slide (Lab-Tek, Nunc, Naperville, Il, USA) overnight. After washing with phosphate-buffered saline (PBS; Sigma-Aldrich Co.), the cells were fixed with Karnovsky’s fixation solution containing 2% paraformaldehyde (Sigma-Aldrich Co.) with 2.5% glutaraldehyde (Sigma-Aldrich Co.) in 0.2 M cacodylate (pH 7.4) (Sigma-Aldrich Co.) for 2 hours at 4 °C, followed by incubation with 1% osmium tetroxide (OsO_4_) buffer for 1.5 hours in the dark for post-fixation, rinsing, dehydration, and embedding. Ultra-thin slices were cut from the dried sections with a diamond knife and placed on the grids. Photographic images were taken using a TEM with a CCD camera (Hitachi H-7100; Hitachi, Ibaraki, Japan).

#### Co-staining with Prussian Blue and phosphotungstic acid haematoxylin (PTAH)

To localize the intracellular ION, 1 × 10^5^ hMSCs were exposed to 100 μg Fe/mL ION (Resovist, 45–60 nm) (Schering AG, Berlin, Germany) for 24 hours and then transferred to NIM and incubated for 2~3 weeks. The hMSCs were treated with a 1:1 mixture of 2% potassium ferrocyanide (Prussian Blue) and 1 M hydrochloric acid for 5 minutes. Furthermore, the hMSCs and NCs were stained for astrocytes, fibroglia and myoglia using PTAH (Sigma-Aldrich Co.) for 20 minutes at room temperature. The cells were then washed twice and imaged using a Nikon TE2000-S inverted microscope.

### Reverse transcription polymerase chain reaction (RT-PCR)

Total RNA from hMSCs and NCs was extracted with TriZol Reagent (Invitrogen, Carlsbad, CA, USA) according to the manufacturer’s instructions. Briefly, the cells were harvested after 21 days of incubation and lysed in supplied lysis buffer. Total RNA concentration was determined by measuring the optical density at 260 nm (OD260) using a spectrophotometer (DU800; Beckman Coulter, Fullerton, CA, USA). One microgram total RNA was used to generate cDNA using a SuperScript III First-Strand cDNA Synthesis System (Invitrogen, Carlsbad, CA, USA).

Polymerase chain reaction (PCR) conditions and cycle numbers for a linear amplification range were determined and optimized. The primers used are shown in Supplement Table 1(S1). The PCR amplification was carried out using PCR Tag Master Mix (Applied Biosystems, Foster City, CA, USA). The thermal profile for PCR was 96 °C for 5 minutes, followed by 40 cycles of 96 °C for 50 s, 59~63 °C for 90 s, and 72 °C for 90 s. The products were examined by electrophoresis in a 2% agarose gel, stained with ethidium bromide and visualized under UV light. Actin was used as a housekeeping gene. The gene expression level was quantitated using the National Institutes of Health (NIH) ImageJ program (National Institutes of Health, USA). The expression level of housekeeping gene was defined as 1.0. The expression ratios of hMSC-derived neural specific genes to the housekeeping gene were determined.

### Western blotting

HMSC and NC lysates were prepared according to standard procedures. Protein content was quantified using a BCA protein assay kit (Pierce, Rockford, IL, USA). Ten micrograms total protein from each lysate was separated by 10% sodium dodecyl sulphate–polyacrylamide gel electrophoresis (SDS–PAGE) and transferred electrophoretically to a polyvinylidene fluoride (PVDF) transfer membrane (NEN Life Science Products, Boston, MA, USA). All membranes were blocked with 5% nonfat dry milk in PBS for 1 hour at room temperature, then incubated with mouse anti-neuron specific enolase (NSE), anti-GFAP, anti-TH, anti-β-III tubulin (Tuj1) (1:1000; Millipore, Billerica, MA, USA) and anti-actin (1:2000; Millipore, Billerica, MA, USA). After washing with PBST, the membrane was incubated with horseradish peroxidase-conjugated secondary antibodies (human anti-mouse IgG, 1:10,000; Millipore, Billerica, MA, USA) diluted in PBS with 5% nonfat milk and 0.1% Tween-20 (PBST) for 2 hours at room temperature. The membranes were visualized using enhanced chemiluminescence (ECL Western blotting detection reagents; Amersham Pharmacia Biotech, Piscataway, NJ, USA). Actin was used as an internal control.

### Immunofluorescence staining

HMSCs were plated for 24 hours in 48-well culture plates at 2.5 × 10^3^ cell/wells before the experiment. After incubation with or without ION for 24 hours, the hMSCs were cultured in CM, and NCs were cultured in NIM for 21 days. The cells were then washed three times with PBS before fixing in 4% paraformaldehyde solution (Sigma-Aldrich) in PBS at room temperature for 10 minutes. The cells were then washed twice with PBS and permeabilized with 0.1% Triton X-100 (Sigma-Aldrich) for 5 minutes. Nonspecific binding sites were blocked using a 2% BSA solution for 30 minutes at room temperature. hMSCs were incubated with neural primary antibodies including GFAP (1:50) for astrocytes, NeuN (1:100), TH (1:500) and Tuj1 (1:200) overnight at 4 °C. The cells were washed and then incubated with fluorescent (FITC/Rhodamine) secondary anti-mouse or anti-rabbit IgG antibodies (Millipore) at room temperature for 45 minutes. The platelets were washed again with PBS, follow by staining with DNA binding dye, 4′,6-diamidino-2-phenylindole (DAPI; 5 μg/mL; Molecular Probes) in PBS for 5 minutes at room temperature. The cells were then washed twice and imaged using an inverted microscope (Eclipse TS100; Nikon, Tokyo, Japan).

### Flow cytometry analysis of neural markers

hMSCs were plated 24 hours before the experiment in 6-well culture plates at 1 × 10^5^ cell/well. After incubation with or without ION for 24 hours, the hMSCs were trypsinized and washed three times with PBS. The cells were fixed in 4% paraformaldehyde solution in PBS at room temperature for 10 minutes, washed twice with PBS and permeabilized with methanol at 4 °C for 15 minutes. The non-specific binding sites were blocked with 2% BSA solution at room temperature for 30 minutes. After centrifugation at 1,500 rounds per minute (rpm) for 5 minutes at 4 °C, the cells were re-suspended in PBS. After washing, the cells were labelled with one of the following primary antibodies: GFAP (1:50), NeuN (1:100), TH (1:500) and Tuj1 (1:200) in 2% BSA solution at 4 °C overnight. After washing, the cells were further incubated with secondary fluorescein isothiocyanate (FITC)-conjugated anti-mouse or anti-rabbit IgG (Millipore) at room temperature for 45 minutes. The platelets were washed with PBS and then collected for FL1 measurement. For each group of stem cells, 1,500 counts were measured via FACS Calibur flow cytometry (FACS Calibur; BD Biosciences, Franklin Lakes, NJ, USA) and CellQuest Pro software (Becton Dickenson, Mississauga, CA).

### Electrophysiological recording

A multi-electrode recording MED64 system (Alpha Med Scientific, Japan) was used to observe the changes in hMSC-derived nerve action potentials. Each MED probe contained 64 electrodes in an 8 × 8 grid set point (Alpha Med Science, MED-P515A) and was coated with 5 μg/mL poly-lysine/laminin (Sigma-Aldrich, St. Louis, MO, USA) at 37 °C in a 5% CO_2_, 95% air atmosphere for 2 hours. After washing with ddH_2_O, 1 × 10^5^ of cells were seeded onto the MED probes and incubated at 37 °C in a 5% CO_2_, 95% air atmosphere overnight. CM and NIM were changed every 2 days for 3 weeks. The multi-electrode recordings used a sampling rate of 50 kHz. Total spikes were counted, and the frequency was analysed with a spike sorting analysis system.

### Flow cytometry detection of ION particle uptake

hMSCs were plated 24 hours before the experiment in 6-well culture plates at 1 × 10^5^ cell/well. After incubation with or without ION for 24 hours, CM and NIM were changed every 2 days for 3 weeks. hMSCs were trypsinized and washed three times with PBS. ION particle uptake by the cells was determined by the number of SSC measurements. For each group of stem cells, 1,500 cell counts were measured via FACS Calibur flow cytometry (FACS Calibur; BD Biosciences, Franklin Lakes, NJ, USA) and CellQuest Pro software (Becton Dickenson, Mississauga, CA).

### Magnetic resonance imaging (MRI)

MRI was performed using a 7 Tesla animal MR system (Bruker Biospec, 70/30, USR). After ION labelling, undifferentiated and differentiation cells in 6-well plates (1 × 10^5^ cells per well) were collected by trypsinization and then washed, centrifuged, and placed in 300-μl Eppendorf tubes (1 × 10^5^ cells per tubes) in a water tank. T2-rapid acquisition with relaxation enhancement (RARE) pulse sequences were used (TR/TE = 3000/12.276 ms, flip angle = 180°, matrix size = 256 × 256). The slice thickness was 1.0 mm with a 1.0-mm gap. The field of view (FOV) was 80 × 80 mm for coronal scanning of the test tubes and 10 minutes and 40 s for sagittal scanning at the NEX of 5. All images were then analysed using the Import Bruker NMR Files and ImageJ software (http://rsb.info.nih.gov/ij/plugins/bruker.html).

### Intracellular iron content determination

The total uptake of Fe by cells was analysed using ICP-MS (Agilent 7500ce, Agilent Technologies, Palo Alto, CA, USA). 1 × 10^5^ cells treated with or without 100 μg Fe/mL ION were collected after 24 hours. The cells were trypsinized, washed and centrifuged. Cell pellets were lysed with 1 ml of 3% HNO_3_ (65% HNO_3_) acid solution. Samples (10 µl) were diluted in 10 ml acid solution and injected to ICP-MS. The Fe concentration was obtained by interpolating to a standard curve obtained from serial dilutions of 0, 10, 20, 50 and 100 ppb Fe.

### Viability assay

Cell viability was evaluated by 0.5 mg/mL MTT (3-(4,5-dimethylthiazol-2-yl)-2,5- diphenyltetrazolium bromide; Sigma-Aldrich Co.) dye assay. Following ION labelling, hMSCs, NCs and unlabelled cells were grown in triplicate in 24-well plates overnight (2.5 × 10^4^ cells/well). Afterward, MTT dye was added to the medium. and the cells were incubated for 1 hour. After incubation, the purple formazan dye generated by viable cells was proportional to the number of viable cells, and absorbance at 570 nm was measured using a microplate reader (Infinite F200; TECAN., Austria).

### Reactive oxygen species measurements

The presence of ROS is an important indicator of oxidative stress. The intracellular ROS was evaluated using dichlorofluorescein diacetate (DCFDA) (Molecular Probes, Eugene, OR, USA) as an oxidation fluorescent probe. 1 × 10^5^ hMSCs or NCs were labelled with 100 μg/mL ION for 24 hours. Cells were washed twice with PBS and incubated in 6-well plates at 37 °C for 21 days. Each group was then washed three times with PBS and incubated in culture medium with 10 µM DCFDA at 37 °C in the dark for 30 minutes. Cells were then collected and re-suspended in cold PBS. The fluorescent mean intensity (FL-1) of positive cells was analysed using a FACS Calibur flow cytometer (Becton-Dickison, PharMingen USA) with an excitation wavelength of 488 nm and an emission wavelength of 515 nm.

### Mitochondria membrane potential measurements

Mitochondrial membrane energization was determined by a lipophilic cationic fluorochrome dye, 3,3′-dihexyloxacarbocyanine iodide [DiOC6 (3)] method (Sigma-Aldrich Co).

Briefly, hMSCs and NCs were treated with 40 nM DiOC6(3) in culture medium and then incubated for 30 minutes at 37 °C in the dark. Cells were collected and washed in cold PBS. The fluorescent mean intensity was analysed using a flow cytometer with an excitation wavelength of 488 nm and an emission wavelength of 501 nm.

### Statistical analysis

Data were analysed using SPSS statistical software (Version 12.0; IBM Corporation, Armonk, NY, USA). One-way repeated measures ANOVA and Dunnett’s t-test were used to compare the means. A p value less than 0.05 was considered statistically significant.

## Electronic supplementary material

Supplementary 1 (S1)
