# Supplementary material for: Characterization of an iron oxide nanoparticle labelling and MRI-based protocol for inducing human mesenchymal stem cells into neural-like cells
Source: Sci Rep. 2017 Jun 15;7:3587. doi: 10.1038/s41598-017-03863-x (PMC5472606; doi:10.1038/s41598-017-03863-x)
Supplement: Supplementary file 1 — Supplementary 1 (S1) [file 41598_2017_3863_MOESM1_ESM.doc]

**Characterization of an iron oxide nanoparticle labelling and MRI-based protocol for inducing human mesenchymal stem cells into neural-like cells**

*Chen-Wen Lu1, 2, Jong-Kai Hsiao2, Hon-Man Liu^3^,*********, Chung-Hsin Wu^1^,********

*1 Department of Life Science, National Taiwan Normal University, Taipei, 10677, Taiwan.*

*2 Department of Medical Imaging, Taipei TzuChi Hospital, The Buddhist TzuChi Medical Foundation, New Taipei*

*City 23142, Taiwan.*

*3 Department of Medical Imaging, National Taiwan University Hospital, Taipei, 10048, Taiwan.*

**Correspondence to Hon-Man Liu, hmliu@ntu.edu.tw; Chung-Hsin Wu, megawu@ntnu.edu.tw*

**Supplementary Table S1**

**Primer sequences used for RT-PCR analysis**

| **Primer** | **Forward (5'- 3')** | **Reverse (5'- 3')** | **Hybridization**  **Temperature**  **(°C)** | **Cycles** | **Amplicon size (bp)** |
| --- | --- | --- | --- | --- | --- |
| **GFAP** | **gtg gta ccg ctc caa gtt tgc ag** | **aat ggt gat ccg gtt ctc ctc** | **59** | **40** | **373** |
| **NEUROD6** | **ctg aga atc ggc aag aga cc** | **ctg cac agt aat gca tgc cg** | **62** | **35** | **433** |
| **TH** | **atc cac cat cta gag acc cg** | **tcc ccg ttc tgc tta cac ag** | **63** | **40** | **824** |
| **NSE** | **aag gac aaa tac ggc aag ga** | **tgg acc agg cag ccc aat c** | **55** | **30** | **328** |
| **actin** | **ggc ggc acc acc atg tac cct** | **agg ggc cgg act cgt cat act** | **55** | **30** | **200** |
